# Prospective controlled study on the effects of deep brain stimulation on driving in Parkinson’s disease

**DOI:** 10.1038/s41531-023-00545-6

**Published:** 2023-07-03

**Authors:** Odette Fründt, Tina Mainka, Eik Vettorazzi, Ela Baspinar, Cindy Schwarz, Martin Südmeyer, Christian Gerloff, Wolfgang H. Zangemeister, Monika Poetter-Nerger, Ute Hidding, Wolfgang Hamel, Christian K. E. Moll, Carsten Buhmann

**Affiliations:** 1 Department of Neurology, University Medical Center Hamburg-Eppendorf, 20246 Hamburg, Germany; 2 Department of Neurology, Ernst von Bergmann Clinic, 14467 Potsdam, Germany; 3 Department of Neurology with Experimental Neurology, Charité Campus Mitte, 10117 Berlin, Germany; 4 BIH Charité Clinician Scientist Program, Berlin Institute of Health at Charité Universitätsmedizin Berlin, Berlin, Germany; 5 Department of Medical Biometry and Epidemiology, University Medical Center Hamburg-Eppendorf, 20246 Hamburg, Germany; 6 Department of Neurosurgery, University-Hospital Hamburg-Eppendorf, 20246 Hamburg, Germany; 7 Institute of Neurophysiology and Pathophysiology, University-Hospital Hamburg-Eppendorf, 20246 Hamburg, Germany

**Keywords:** Parkinson's disease, Parkinson's disease

## Abstract

To explore the influence of bilateral subthalamic deep brain stimulation (STN-DBS) on car driving ability in patients with Parkinson’s disease (PD), we prospectively examined two age-matched, actively driving PD patient groups: one group undergone DBS-surgery (PD-DBS, *n* = 23) and one group that was eligible for DBS but did not undergo surgery (PD-nDBS, *n* = 29). In PD-DBS patients, investigation at Baseline was done just prior and at Follow-up 6–12 month after DBS-surgery. In PD-nDBS patients, time interval between Baseline and Follow-up was aimed to be comparable. To assess the general PD driving level, driving was assessed once in 33 age-matched healthy controls at Baseline. As results, clinical and driving characteristics of PD-DBS, PD-nDBS and controls did not differ at Baseline. At Follow-up, PD-DBS patients drove unsafer than PD-nDBS patients. This effect was strongly driven by two single PD-DBS participants (9%) with poor Baseline and disastrous Follow-up driving performance. Retrospectively, we could not identify any of the assessed motor and non-motor clinical Baseline characteristics as predictive for this driving-deterioration at Follow-up. Excluding these two outliers, comparable driving performance between PD-DBS and PD-nDBS patients not only at Baseline but also at Follow-up was demonstrated. Age, disease duration and severity as well as Baseline driving insecurity were associated with poorer driving performance at Follow-up. This first prospective study on driving safety in PD after DBS surgery indicates that DBS usually does not alter driving safety but might increase the risk for driving deterioration, especially in single subjects with already unsafe driving prior to DBS surgery.

## Introduction

Patients with Parkinson’s disease (PD) frequently depend on car driving due to impairments of mobility and gait. About 60% of all Parkinson’s disease patients and 50% of those with subthalamic deep brain stimulation (STN-DBS) actively drive a car^1^. Generally, PD patients drive less safely^2,3^ and quit driving more often than controls^4^. Age and cognitive impairment are the main risk factors for impaired driving^5^ while slight to moderate motor impairment plays a minor role (reviews^5–7^:). Previously, we illustrated in a controlled cross-sectional driving simulator study that PD patients with subthalamic DBS (STN-DBS) drive safer than patients without DBS despite comparable age but even higher disease severity according to Hoehn & Yahr. Furthermore, DBS patients drove better in the test condition “stimulation on” than with “medication on” alone, despite a comparable positive effect of both conditions on motor disability^8^. This suggests potential beneficial effects of subthalamic DBS on driving ability besides motor but potentially due to non-motor driving relevant aspects.

However, there is no investigation yet assessing the influence of STN-DBS on driving in PD patients in a controlled and prospective manner. Due to the increasing number of DBS-treated PD patients it is clinically relevant to know whether STN-DBS influences driving safety. As motor impairment is not crucial for driving ability in both PD patients with^8^ and without STN-DBS^2,9^, other factors found to improve after DBS, such as implicit procedural learning, sequence learning, goal-directed action selection or decision-learning (review^10^) might be more relevant skills when driving a car. On the other hand, STN-DBS can induce cognitive impairment with a decline of executive functions^11^ or altered impulse control^12^, both of which can interfere with driving ability.

Here, we applied an evidence class II study design to prospectively evaluate driving performance in PD patients before and six to twelve months after STN-DBS implantation (depending on the moment of reaching a stable postoperative stimulation and medication adjustment). Driving performance was also compared to a group of DBS-eligible but non-operated PD patients under best medical treatment that was frequency-matched for age, cognition, and disease severity. Assessment of driving performance in controls at one time-point should allow estimating the PD patients’ general level of driving ability.

The aim of this single-centre study was to explore the potential effect of DBS on driving ability and safety within 1 year post-surgery.

## Results

A total of 128 subjects were screened. In total, 85 participants (23 PD-DBS, 29 PD-nDBS, 33 controls) were included into the study with complete data sets (two driving sessions each at Baseline and Follow-up in 60 study participants and at least one driving session at Baseline and Follow-up in 25 participants). DBS was done in all cases bilaterally. All patients were in the clinical “on”-state during driving and scoring assessments and did not show any disabling dyskinesia.

Supplementary Table 1 informs on drop-out reasons of the excluded 43 of 128 screened subjects, Supplementary Table 2 describes reasons of the 25 included patients with less than 4 driving sessions and Supplementary Table 3 presents completion rates per session and group. In the PD-DBS group, Baseline examinations were performed in, on average, 76.3 days (±SD 62.3 [2–231]) prior to DBS implantation. Follow-up sessions of both patient groups were performed about 9.15 months (±SD 2.7 [4–18]) after Baseline (Supplementary Table 4 shows the distribution of Follow-up dates). Time to Follow-up differed between the PD-DBS (10.4 months +/- SD 3.0 [7–18]) and PD-nDBS (8.2 months ±SD 2.0 [4–11], *p* = 0.003) group.

### Clinical scores and questionnaires

Baseline clinical characteristics of all groups are given in Table 1. All three groups did not differ significantly regarding age, sex/gender (self-reported), disease duration, driving experience, and cognition according to MMSE. Healthy controls scored higher in the PANDA and lower in the BDI and PDQ-39 compared to both patient groups (all *p* < 0.005) indicating less cognitive impairment and depressed mood and better quality of life in healthy subjects, respectively. Importantly, clinical characteristics, especially motor scores (H&Y and UPDRS III), cognition (MMSE and PANDA) and medication (total LED of anti-parkinsonian medication as well as LED and frequency of use of dopamine agonists) did not differ significantly between PD-DBS and PD-nDBS group at Baseline (Table 1).

**Table 1:** Baseline clinical parameters of all study participants.

Clinical parameter	PD-DBS (*n* = 23) Mean +/- SD, Min-Max	PD-nDBS (*n* = 29) Mean +/- SD, Min-Max	Controls (*n* = 33) Mean +/- SD, Min-Max	Statistics
Age (years)	60.1 (±SD 8.1; 45–72)	61.4 (±SD 7.6; 49–73)	57.7 (±SD 11.3; 29–77)	F(2,82) = 1.230, *p* = 0.298
Sex/Gender (n; female/male)	7 /16	5/24	12/21	χ²(2) = 2.875, *p* = 0.238^a^
Disease duration (years)	11.0 (±SD 5.0; 5–27)^b^	9.0 (±SD 4.8; 2–24)	n.a.	F(1,49) = 2.017, *p* = 0.162
Ownership of driver license (years)	41.3 (±SD 10.2; 20–58)	41.1 (±SD 7.8; 26–55)	37.7 (±SD 11.7; 11–58)	F(2,82) = 1.225, *p* = 0.299
Kilometres driven during the last 3 years (km)	31822 (±SD 25232; 300–80000)	34293(±SD 59797; 1000–30000)	41424 (±SD 42567; 2000–20000)	F(2,82) = 0.343, *p* = 0.710
Subjective Driving Safety (n)	- safe: 11 - average: 10 - unsafe: 2	- safe: 15 - average: 11 - unsafe: 3	- safe: 20 - average: 11 - unsafe: 2	χ²(4) = 1.179, *p* = 0.882^a^
Accidents during last 3 years (n)	- 1 accident: 3 - >1 accident: 0	- 1 accident: 8 - >1 accident: 0	- 1 accident: 3 - >1 accident: 2	χ²(4) = 7.006, *p* = 0.136
Levodopa Equivalent Dose (LED, mg)	1,169.8 (±SD 594.6; 152–2,740)	1,070.6 (±SD 455.1; 210–1,812)	n.a.	F(1,50) = 0.465, *p* = 0.498
Dopamine agonists – LED (mg)	288.7 (±SD 219.2; 0–880)	347.3 (±SD 247.5; 0–1280)	n.a.	F(1,50) = 0.794, *p* = 0.377
Dopamine agonists – frequency of use (n)	20	28	n.a.	χ²(1) = 1.663, *p* = 0.197^a^
Hoehn & Yahr Score	2.2 (±SD 0.8; 0–4)	2.3 (±SD 0.6; 1–3)	n.a.	F(1,50) = -0.225, *p* = 0.823
UPDRS III	16.8 (±SD 11.4; 1.5–43)	16.4 (±SD 8.5; 3.5–39.5)	n.a.	F(1,50) = 0.170, *p* = 0.866
MMSE (screening exclusion criterion)	28.2 (±SD 3.2; 15–30)^c^	28.7 (±SD 1.9; 24–30)	28.8 (±SD 1.5; 24–30)	F(2,82) = 6.435, *p* = 0.514
PANDA	20.4 (±SD 6.5; 9–30)^d^	20.2 (±SD 6.0; 5–28)	24.8 (±SD 4.2; 14–30)	F(2,81) = 6.624, *p* = 0.002**
BDI	9.4 (±SD 6.8; 1–30)	7.0 (±SD 5.2; 1–25)	2.2 (±SD 2.8; 0–13)^e^	F(2,81) = 15.232, *p* < 0.001***
PDQ-39	30.4 (±SD 18.1; 6.4–82.0)	25.1 (±SD 11.8; 5.1–53.2)	2.2 (±SD 3.5; 0–12.8)^f^	F(2,80) = 44.244, *p* < 0.001***

Clinical parameters and scores at Baseline are shown for the three groups as mean (with standard deviation SD; minimum and maximum). Statistics of univariate ANOVA (F-value, degree of freedom, *p* value) are given in the last column.

*n.a.* not applicable, *n* number of participants, *mg* milligram.

^a^Analyzed with Pearson chi-square test (χ²-value, degree of freedom, *p* value).

^b^Data of 1 participant missing (disease duration).

^c^One patient scored below the MMSE cut-off (against clinical impression), but extended neuropsychological testing was unremarkable without evidence for manifest dementia.

^d^Data of 1 participant not documented (PANDA).

^e^Data of 1 participant not documented (BDI).

^f^Data of 2 participants not documented (PDQ-39).

At Follow-up (Table 2) lower (better) H&Y scores and numerically lower (better) UPDRS III scores in PD-DBS compared to PD-nDBS participants were found (*p* = 0.022 and *p* = 0.064, respectively). Total LED (*p* = 0.001), dopamine agonist LED (*p* < 0.001) and frequency of their use (*p* = 0.032) were also lower in the PD-DBS compared to the PD-nDBS group. Other clinical parameters at Follow-up did not differ significantly.

**Table 2:** Follow-up clinical parameters of all study participants.

Clinical parameter	PD-DBS (*n* = 23) Mean ± SD [Min-Max]	Number of patients with available data (PD-DBS)	PD-nDBS (*n* = 29) Mean ± SD [Min-Max]	Number of patients with available data (PD-nDBS)	Statistics
Levodopa Equivalent Dose (LED, mg)	661.1 ± 353.9 [0–1322.5]	*n* = 23	1111.4 ± 504.0 [75–1948.8]	*n* = 24	t(45) = 12.466, *p* = 0.001**
Dopamine agonists—LED (mg)	105.0 (±SD 118.6; 0–484.8)	*n* = 23	309.7 ( ±SD 205.4; 0–800)	*n* = 24	t(45) = 17.318, *p* < 0.001***
Dopamine agonists—frequency of use (n)	15	*n* = 23	22	*n* = 24	χ²(1) = 4.600, *p* = 0.032*^a^
Hoehn & Yahr Score	2.1 ± 0.5 [1–3]	*n* = 23	2.4 ± 0.4 [1.25–3]	*n* = 29	t(50) = –2.369, *p* = 0.022*
UPDRS III	14.9 ± 10.3 [4–47]	*n* = 23	20.2 ± 9.7 [8–48]	*n* = 29	t(50) = –1.894, *p* = 0.064
PANDA	20.7 ± 6.0 [9–29]	*n* = 23	22.7 ± 4.6 [11–28]	*n* = 29	t(50) = –1.328, *p* = 0.190
BDI	6.8 ± 4.1 [1–16]	*n* = 20	6.6 ± 4.9 [0–20]	*n* = 22	t(40) = 0.185, p = 0.854
PDQ_39	27.3 ± 12.7 [4.2–46.5]	*n* = 17	24.8 ± 13.4 [10.9–52.8]	*n* = 21	t(36) = 0.587, *p* = 0.561
Time from Baseline to Follow-up (month)	10.4 ± 3.0 [7–18]	*n* = 23	8.2 ± 2.0 [4–11]	*n* = 29	t(50) = 3.090, *p* = 0.003**

Clinical parameters and scores at Follow-up are shown for both the PD groups as mean (standard deviation SD; minimum and maximum). Statistics of *t* tests are given in the last column as t-value, degree of freedom and *p* value.

*n* number of participants.

^a^Analyzed with Pearson chi-square test (χ²-value, degree of freedom, *p* value).

### Group comparisons of clinical changes from Baseline to Follow-up

Clinical changes from Baseline to Follow-up were more pronounced in the PD-DBS compared to the PD-nDBS group, with decreased LED (*p* < 0.001), LED of dopamine agonists (*p* < 0.001) and H&Y scores (*p* = 0.027), indicating less need for medication, reduced agonist dosages and better clinical condition after DBS-surgery. All other clinical scores (UPDRS III, PANDA, BDI, PDQ-39) did not show any significant group differences when comparing Baseline vs. Follow-up (Supplementary Table 5).

### Driving simulator performance

#### Error rates

Table 3 shows the detailed measurements of driving performance at Baseline and Follow-up investigation for healthy controls and both PD patient groups. Statistic values are given for between-group comparisons at Baseline and Follow-up (left part of the table) as well as for within-group comparisons from Baseline to Follow-up (right part).

**Table 3:** Driving performance parameters of all participants.

					Between group comparisons				Within group comparisons			
Parameter	Time point	Controls (*n* = 33)	PD-DBS (*n* = 23)	PD-nDBS (*n* = 29)	PD vs Ctrls	*p* value	DBS vs nDBS	*p* value	FU vs BL: DBS	*p* value	FU vs BL: nDBS	*p* value
**Driving time** (in s; estimated residuals, LMM)	Baseline	835.0 (787.5, 882.5)	852.4 (795.5, 909.4)	856.9 (806.2, 907.7)	19.68 (–41.26, 80.62)	0.524	–4.54 (–80.79, 71.71)	0.906	–	–	–	–
	Follow-up	–	825.7 (768.7, 882.6)	831.6 (779.1, 884.1)	–	–	–5.94 (–83.37, 71.50)	0.880	-26.76 (-97.96, 44.43)	0.458	-25.36 (-90.18, 39.47)	0.440
**Absolute Error rate** (mean, estimated residuals, LMM)	Baseline	15.8 (13.6, 18.5)	19.0 (15.9, 22.7)	18.9 (16.1, 22.1)	1.20 (0.99, 1.45)	0.069	1.01 (0.79, 1.28)	0.949	–	–	–	–
	Follow-up	–	20.8 (17.4, 24.8)	17.4 (14.8, 20.4)	–	–	1.20 (0.94, 1.52)	0.136	1.09 (0.93, 1.28)	0.274	0.92 (0.79, 1.07)	0.264
**Error severity** (quotients, gLMM)												
- Slight	Baseline	9.4 (8.2, 10.8)	9.7 (8.2, 11.4)	10.1 (8.8, 11.7)	1.06 (0.89, 1.26)	0.536	0.96 (0.77, 1.19)	0.702	–	–	–	–
	Follow-up	–	10.0 (8.5, 11.7)	8.9 (7.7, 10.4)	–	–	1.12 (0.90, 1.39)	0.318	1.03 (0.85, 1.24)	0.768	0.88 (0.75, 1.05)	0.149
- Moderate	Baseline	3.6 (2.8, 4.7)	6.3 (4.7, 8.3)	5.3 (4.1, 6.9)	1.60 (1.16, 2.21)	0.005	1.18 (0.80, 1.72)	0.401	–	–	–	–
	Follow-up	–	6.8 (5.1, 9.0)	5.5 (4.2, 7.1)	–	–	1.25 (0.86, 1.82)	0.248	1.09 (0.86, 1.36)	0.474	1.02 (0.82, 1.27)	0.830
- Severe	Baseline	1.8 (1.4, 2.4)	1.5 (1.1, 2.1)	1.7 (1.3, 2.3)	0.87 (0.62, 1.21)	0.396	0.88 (0.57, 1.36)	0.561	–	–	–	-–
	Follow-up	–	1.8 (1.3, 2.5)	1.5 (1.1, 2.0)	–	–	1.22 (0.79, 1.87)	0.365	1.22 (0.77, 1.92)	0.394	0.88 (0.58, 1.33)	0.535
- Very severe	Baseline	0.7 (0.4, 1.1)	1.1 (0.6, 1.8)	1.3 (0.8, 2.0)	1.71 (0.95, 3.09)	0.073	0.85 (0.43, 1.68)	0.642	–	–	–	–
	Follow-up	–	1.7 (1.1, 2.7)	1.0 (0.6, 1.5)	–	–	1.77 (0.92, 3.41)	0.089	1.58 (0.94, 2.67)	0.086	0.76 (0.47, 1.24)	0.273
**Error classes** (quotients, gLMM)												
- Distance	Baseline	0.6 (0.2, 2.1)	3.3 (0.9, 12.6)	1.7 (0.5, 5.5)	3.72 (0.85, 16.21)	0.080	2.00 (0.34, 11.92)	0.443	–	–	–	–
	Follow-up	–	1.2 (0.3, 4.6)	1.3 (0.4, 4.4)	–	–	0.88 (0.14, 5.44)	0.894	0.35 (0.05, 2.34)	0.276	0.79 (0.15, 4.33)	0.788
- Indicator	Baseline	4.9 (3.9, 6.1)	6.7 (5.3, 8.5)	6.0 (4.8, 7.5)	1.29 (0.99, 1.68)	0.057	1.11 (0.81, 1.53)	0.514	–	–	–	–
	Follow-up	–	6.0 (4.7, 7.6)	5.1 (4.1, 6.4)	–	–	1.16 (0.84, 1.61)	0.365	0.89 (0.67, 1.18)	0.421	0.85 (0.66, 1.11)	0.228
- Lane keeping	Baseline	8.6 (6.0, 12.4)	14.2 (9.4, 21.4)	13.0 (9.0, 18.8)	1.57 (1.00, 2.47)	0.049	1.09 (0.63, 1.89)	0.758	–	–	–	–
	Follow-up	–	18.7 (12.4, 28.3)	12.3 (8.5, 17.8)	–	–	1.52 (0.88, 2.65)	0.132	1.32 (0.93, 1.88)	0.123	0.94 (0.68, 1.30)	0.724
- Accident	Baseline	2.5 (1.4, 4.5)	3.1 (1.6, 6.1)	3.3 (1.8, 6.0)	1.26 (0.61, 2.61)	0.524	0.95 (0.38, 2.33)	0.902	–	–	–	–
	Follow-up	–	3.0 (1.5, 5.8)	2.8 (1.5, 5.0)	–	–	1.07 (0.43, 2.65)	0.880	0.94 (0.36, 2.45)	0.906	0.83 (0.36, 1.95)	0.672
- Velocity (Speed)	Baseline	9.6 (8.4, 11.0)	8.0 (6.8, 9.6)	8.9 (7.7, 10.4)	0.88 (0.74, 1.05)	0.157	0.90 (0.72, 1.13)	0.364	–	–	–	–
	Follow-up	–	8.4 (7.0, 9.9)	8.7 (7.5, 10.1)	–	–	0.96 (0.77, 1.21)	0.732	1.04 (0.83, 1.31)	0.739	0.97 (0.80, 1.19)	0.793
- Traffic sign	Baseline	3.2 (2.1, 4.7)	3.6 (2.2, 5.7)	5.3 (3.6, 8.0)	1.39 (0.84, 2.30)	0.203	0.67 (0.36, 1.25)	0.210	–	–	–	–
	Follow-up	–	2.4 (1.5, 3.9)	3.4 (2.3, 5.2)	–	–	0.70 (0.36, 1.34)	0.274	0.67 (0.34, 1.32)	0.243	0.64 (0.36, 1.16)	0.139
**Driving safety score** (DSS, quotients, gLMM)	Baseline	31.9 (19.7, 44.1)	43.5 (28.8, 58.1)	42.3 (29.3, 55.4)	10.99 (-4.66, 26.65)	0.167	1.11 (–18.48, 20.70)	0.911	–	–	–	–
	Follow-up	–	59.1 (44.5, 73.7)	37.7 (24.7, 50.7)	–	–	21.40 (1.81, 40.99)	0.033	15.63 (-0.08, 31.34)	0.051	-4.65 (-18.65, 9.34)	0.512

Driving performance parameters are presented for each of the three groups (PD-DBS = PD patients with DBS; PD-nDBS = PD patients without DBS; Ctrls = healthy controls) at Baseline (upper row) and Follow-up (bottom row). Statistic *p* values are given for between-group comparisons at Baseline and Follow-up (left part of the table) as well as for within-group comparison from Baseline to Follow-up investigation (right part of the table). The first two parameters (driving time and absolute error rate) are presented as the estimated marginal means and their differences of the linear mixed model analysis (LMM). Driving safety scores (DSS) are given as differences (gLMM). For all other parameters results are given as back-transformed rates and quotients (relative differences) derived from the mixed negative binominal models.

At Baseline, overall error rates did not differ between both PD patient groups (PD-DBS: 19.0 vs. PD-nDBS: 18.9; *p* = 0.949), but PD patients in general made numerically more errors compared to healthy controls (15.8, *p* = 0.069). At Follow-up, error rates of both PD patient groups were again similar (20.8 vs. 17.4, *p* = 0.136). Within-group analysis also showed comparable error rates between Baseline and Follow-up in both PD groups. However, error rates numerically decreased in the PD-nDBS group at Follow-up but increased in the PD-DBS after DBS surgery but without statistical significance (all *p* > 0.2).

**Fig. 1:**
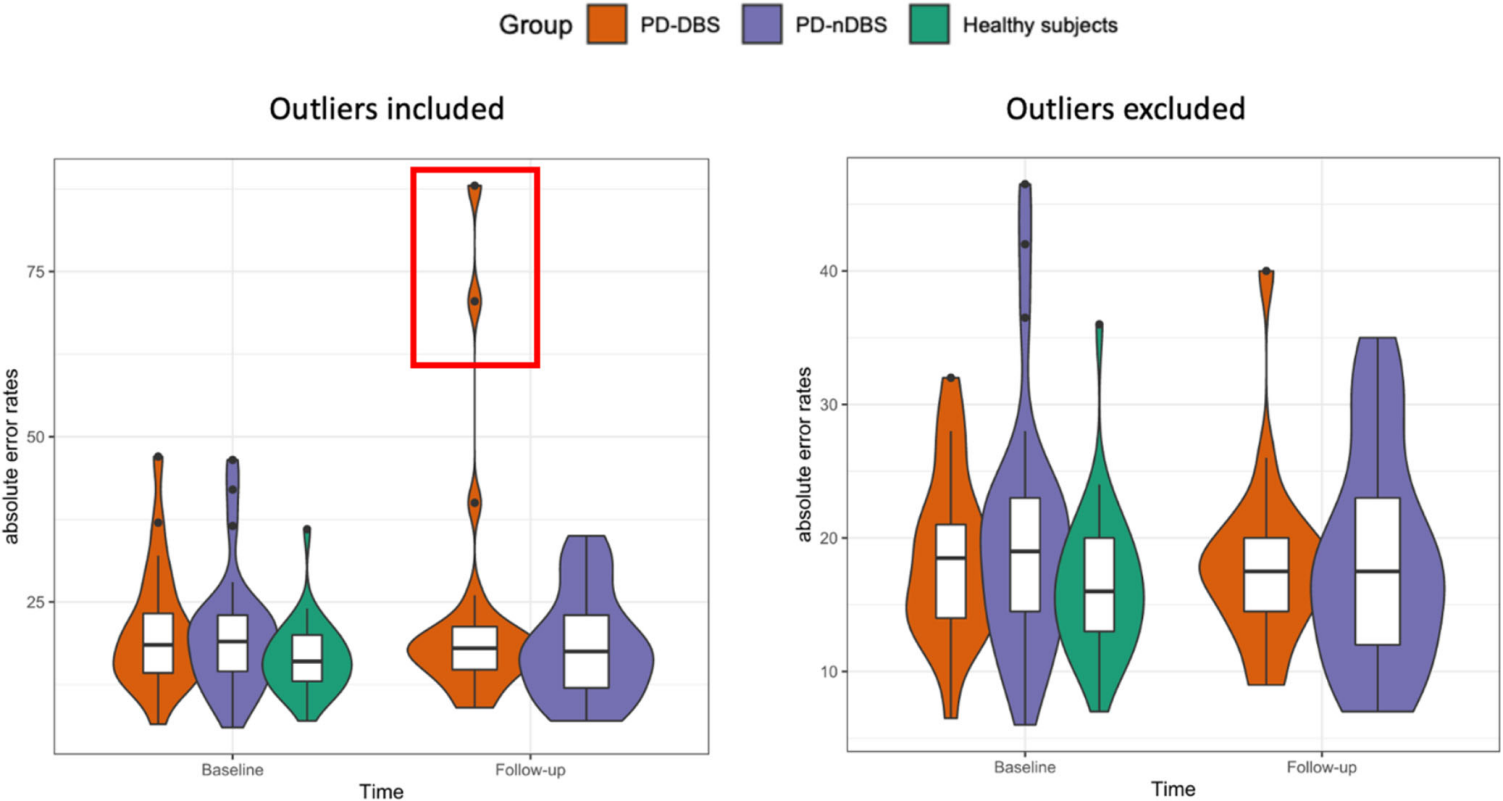
Contrasting juxtaposition of estimated mean absolute error rates of analysis with and without outliers. Chart of distribution of estimated mean absolute errors for each of the three groups including (left) and excluding (right) the outliers: patients with (PD-DBS = orange) and without deep brain stimulation (PD-nDBS = violet) and healthy controls (green) at Baseline and Follow-up. Especially at Follow-up, there are two prominent outliers (red box). Boxplots are given with median, Q1 and Q3 as box margins.

Analyzing error rates, we found two relevant outliers in the PD-DBS group (male and female; Fig. 1, red box) with substantially higher error rates (and DSS) in the post-operative Follow-up session compared to Baseline (88 vs. 37 and 70.5 vs. 47, respectively) and about four-fold higher error rates compared to mean rates of other PD-DBS subjects (19.0 at Baseline and 20.8 at Follow-up). Additionally, both outliers also belonged to the worst five driving performers with the highest overall error rates at Baseline (Supplementary Table 6). However, we kept them included into this main analysis as there were no clinical differences to the other study participants nor obvious external biases (such as technical problems or systematic errors) explaining these results and to reflect “real life” in the sense of an intention to treat concept. Nonetheless, we did a post-hoc analysis excluding these two outliers to estimate their effect on overall results and tried to explore reasons or explaining factors for the strikingly unsafe driving at Follow-up (see below).

#### Error severity

At both time points, most errors of all three participant groups belonged to the “slight error” severity and lowest rates were found for “severe” and “very severe” errors (Table 3). Compared to controls at Baseline, both patient groups showed higher rates of “moderate” and numerically also for “very severe” errors (*p* = 0.005 and *p* = 0.073, respectively). Between-group analyses revealed numerically more “very severe” errors in the PD-DBS (1.7) compared to the PD-nDBS (1.0) group at Follow-up (*p* = 0.089) and within-group comparisons showed a numerically higher rate of “very severe” errors only in the PD-DBS group at Follow-up (1.7) compared to Baseline (1.1; *p* = 0.086).

#### Error category

**Fig. 2:**
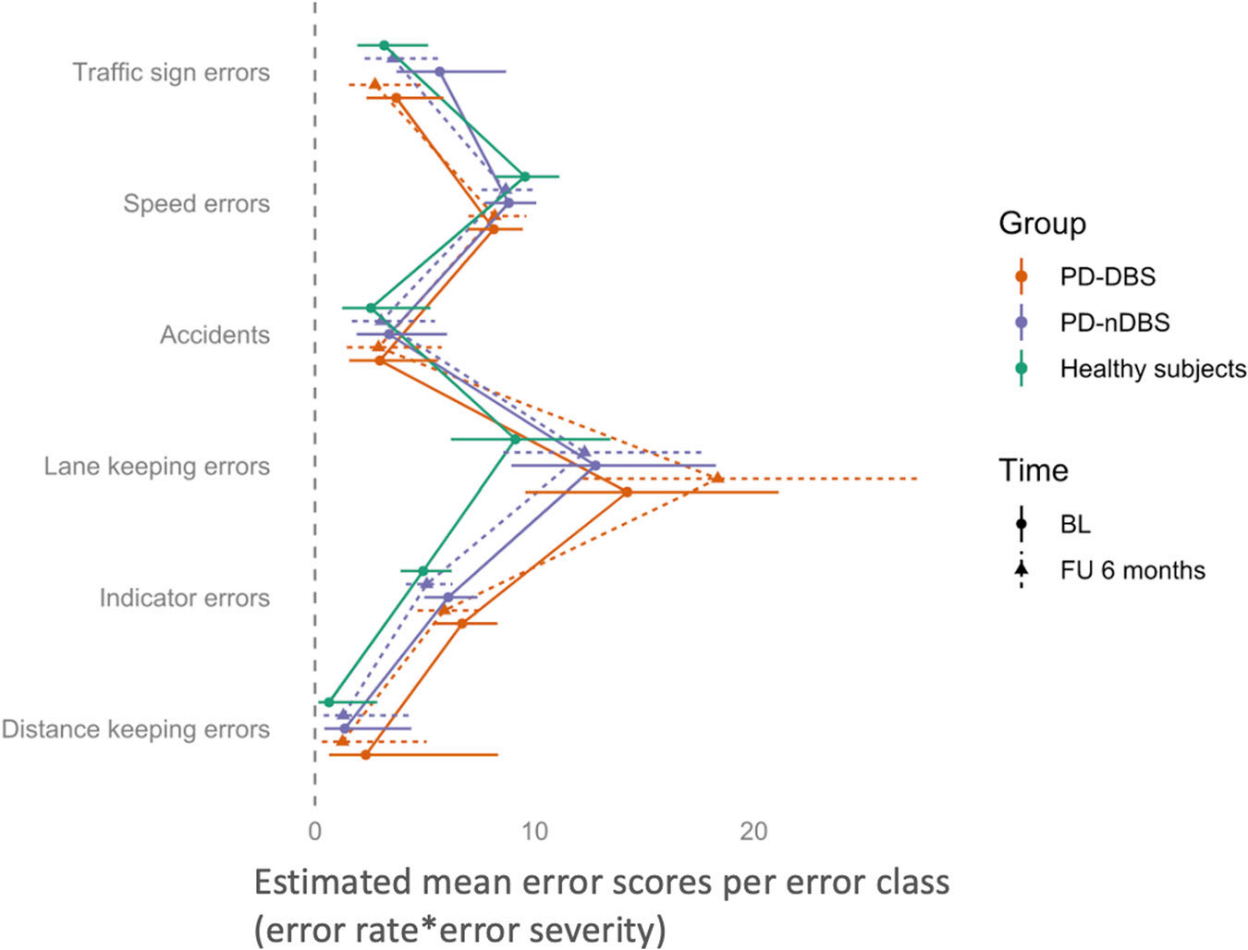
Error scores (error rate*error severity) per error category for all participants. Estimated mean error scores (error rate*error severity) per error category are given for each of the three groups (all participants): patients with (PD-DBS = orange) and without deep brain stimulation (PD-nDBS = violet) and healthy controls (green) at Baseline (solid line) and Follow-up (dotted line). The horizontal bars represent the 95% confidence intervals of the estimated marginal means.

In all three groups, “lane keeping”, “speed”, and “indicator” errors were most common (Table 3). Considering both, error rate and severity, “lane keeping” was the prominent error category in all groups (Fig. 2).

At Baseline, patients with PD made more “lane keeping” (*p* = 0.049) and numerically also “indicator” errors than controls (*p* = 0.057), but both PD groups did not differ significantly regarding error categories at Baseline and Follow-up (between- and within-group comparisons).

### Driving time and driving safety score (DSS)

Driving time at Baseline and Follow-up did not differ between PD patients and controls as well as between both PD patient groups and even regarding within-group comparisons.

At Baseline, PD patients with and without DBS showed a numerically but non-significantly higher DSS compared to controls (43.5 and 42.3 vs. 31.9, respectively with *p* = 0.167), indicating a somewhat poorer driving performance in all PD patients compared to controls. DSS was similar in both PD patient groups at Baseline (*p* = 0.911), but at Follow-up the PD-DBS group showed significantly higher (poorer) DSS scores (59.1) compared to the PD-nDBS groups (37.7, *p* = 0.033) with within-group comparison also revealing a higher (poorer) DSS in the PD-DBS groups’ Follow-up session compared to their Baseline (43.5, *p* = 0.051) which was not found for the PD-nDBS group (*p* = 0.512).

### Variability between test sessions (intraindividual variability)

To address the variability of rater judgments between test sessions (intraindividual variability), we calculated the ICC (intraclass correlation) for the main outcome driving parameters. ICC [95% CI] for DSS was 0.746 [0.628, 0.842] and ICC for error rates was 0.726 [0.613, 0.810].

### Correlations of clinical characteristics with DSS

Spearman correlation analyses of Baseline clinical parameters and DSS reflecting overall driving safety for both PD patient groups combined revealed only weak to moderate correlations (all r < 0.58, Supplementary Table 7) with the strongest correlation of Baseline DSS with the DSS at Follow-up (r = 0.574, CI [0.357, 0.732]), with disease duration (r = 0.356, CI [0.090, 0.575]), age (r = 0.332, CI [0.065, 0.554]) and H&Y (r = 0.382, CI [0.060, 0.551]) indicating that higher age, longer disease duration and greater disease severity are associated with poorer driving performance and that participants who performed worse at Baseline also drove unsafe at Follow-up.

### Post-hoc analysis (outliers excluded)

To estimate the influence and a potential bias of the two PD-DBS outliers on our results, an additional post-hoc analysis of driving performance was done excluding them. Supplementary Tables 8–10 (clinical data at Baseline and Follow-up) and 11 (simulator data at both timepoints) show variations from main analysis marked in yellow for synopsis. We found that these two outliers had significantly influenced the results of the main analysis especially regarding the primary outcome parameter DSS (and the error severity as secondary outcome). Excluding them from analysis, results are as follows (as the outliers lead to more instable modelling and distorted effects, we here only present numerical data without addressing significances (*p*-values) of the results):

Results for clinical scores were similar compared to main analysis at both timepoints and with respect to clinical changes from Baseline to Follow-up (Supplementary Tables 8–10).

Results for driving performance deviated (Supplementary Table 11). While absolute error rates and driving time remained comparable to the main analysis, Baseline DSS is now not only numerically but significantly higher (=poorer performance) in PD patients compared to healthy controls, but again similar between both PD groups. As most relevant difference to the main analysis, post-hoc analysis showed no different but similar driving performance in both PD patient groups, with and without DBS, not only at Follow-up (DSS: 36.5 vs. 37.7) but also with respect to inner-PD group comparisons. This indicates no relevant effects of DBS on driving safety when excluding the two outliners from analysis. Also, PD patients with and without DBS did not differ regarding the rate of “very severe” errors at Follow-up anymore. All other parameters such as error categories or correlations between driving performance and clinical aspects were comparable in both, main and post-hoc analysis (Supplementary Table 12).

To address whether it would have been possible at Baseline to identify these two retrospectively at Follow-up strikingly unsafe and incompetently driving outliers, we reviewed their individual clinical characteristics (Supplementary Table 13), but except a suspicious PANDA (<15; but normal MMSE) in one of the two outliners at Baseline, none of our clinical parameters could have “predicted” poor driving performance of these two outliers at study inclusion.

## Discussion

We evaluated driving-simulator performance in patients with Parkinson’s disease (PD) before and after DBS in a prospective and controlled, three-armed design with the parameters of driving safety score (DSS) and overall mean error rate as primary outcomes.

Our data show that (i) potentially DBS-eligible, non-demented PD patients younger than 75 years drive numerically with more errors but overall comparably safe compared to age-matched healthy controls, that (ii) DBS surgery does not alter driving safety in the vast majority of PD patients but can deteriorate driving safety in single subjects and that (iii) poor and unsafe driving prior to DBS operation rather than certain clinical patient characteristics might be a “predictive risk factor” diminishing driving safety postoperatively.

Our methodological approach using a driving simulator assessment permits faithful replication of the experimental road conditions across participants, in contrast to on road settings^13^. Simulator performance reflects real life driving ability^14^ and is suitable to monitor on-road driving impairments in patients with Parkinson’s disease^2^. Assessing speed and driving errors in key safety traffic situations has been approved to reflect safety-related parameters in traffic simulation models^15^. Our application of a self-developed safety score (DSS) weighs for the “quality” of driving errors and accounts for high rate/low severity and low rate/high-severity errors. This is meaningful for determining crash risk in Parkinson’s disease, because especially the latter errors lead to car crashes^13^. Our PD patient groups were very comparable due to selection regarding principle eligibility for DBS. For detailed matching we focused on age and cognition considering that higher age and cognitive impairment are main risk factors for unsafe driving^5^. The controlled prospective study design allows comparison of driving between both patient groups irrespective of the disease progression. On the other hand, Follow-up investigation on average nine months later is likely short enough to rate disease progression as irrelevant anyway but is long enough to reach a stable clinical condition of DBS and/or medication adjustments^16^. The Baseline comparison with age-matched healthy controls enables quantifying the driving level of patients with PD in general. In the healthy controls, we abstained from Follow-up investigations as no relevant change in driving performance was assumed within nine months and without intervention. Despite a mean Follow-up investigation at nine months, time points varied from four to 18 months due to clinical or logistic reasons, resulting in a significant between-patient group difference at Follow-up of about two months. However, this short period should not bias results as it likely does not relevantly affect disease progression between groups.

As a general result, we found healthy controls to drive numerically less erroneous than both PD patient groups. This is in line with former studies showing patients with PD driving somewhat insecurely compared to controls^2,14^. In our study, longer disease duration and higher disease severity (according to H&Y) influenced driving safety negatively, which corresponds to higher accident rates found to be related to disease burden in PD^14,15^. Driving experience (expressed as the driven mileage during the last three years) had no relevant influence on driving safety, which is also in line with former findings^17^. Age and cognitive impairment correlated rather weakly with unsafe driving in our study but strongly in others^2,15,18^. This is likely related to the fact that we only included DBS-eligible patients without relevant cognitive impairment and of comparatively “young” age.

Own previous studies suggested that DBS might influence driving in PD positively: In a survey, patients who had undergone DBS reported three times more subjective improvement than deterioration of driving after DBS operation and postoperatively twice as many patients restarted than quit driving, independently of their H&Y stage^1^. In a previous controlled, cross-sectional class IV driving-simulator study we found a positive effect of DBS on driving when comparing two groups of patients, with and without DBS, and comparing the therapeutic “stimulation on” and the “medication (levodopa) only” condition in the DBS group. Because motor aspects could not explain results, we hypothesized at that time that DBS might improve driving due to non-motor driving relevant cognitive aspects^8^ such as improvements of implicit procedural^19^ and/or sequence learning^20^, goal-directed action selection^21^ or decision-learning^22^; all crucial cognitive skills for car driving.

However, results of the present study do not support this assumption. DBS intervention might even have a negative effect on driving safety, at least in single PD patients. Poorer driving safety (DSS) at Follow-up in the patient group undergone DBS was driven by two prominent bad performing outliers (9%). Excluding them from analysis revealed no changes in driving safety in patients undergone DBS compared to patients who did not. However, because these two outliers still drove a car and could not have been identified as potential unsafe drivers preoperatively with view to clinical data - neither prospectively nor retrospectively - we kept them included in our main analysis to reflect the “real-life” situation.

Interestingly, in contrast to the DSS score, developed by us to weight for error severity and rate, pure error rates were similar between PD patients with and without DBS in the Follow-up, indicating that the severity of driving errors plays a relevant role here. Consecutively, the negative effect on driving can be explained by the occurrence of numerically more “very severe” errors in the PD-DBS group.

Therefore, DBS might lead to a deterioration of driving in certain patients with Parkinson’s disease that, however, do not present with obvious “predicting” clinical characteristics prior to surgery. However, the two outliers belonged to the “worst five” of bad drivers at study inclusion. Hence, unsafe driving prior to surgery might be a risk factor for postoperative deterioration—a hypothesis that is also supported by the results of our correlation analysis. For clinical practice, it seems advisable to recommend such patients particularly urgently to have their driving ability checked not only before but especially after DBS surgery. Alarmingly, in retrospective none of the here assessed clinical parameters and in previous studies described risk factors for driving, such as age, cognition or disease severity according to UPDRS III nor other parameters such as LED indicated these patients’ bad driving or worsening of driving after DBS. We therefore checked all available medical records of both outliers with respect to clinical, surgical and programming specifics (see Supplementary Table 13) and found three clinical similarities, which might be of interest in this context. Both patients had complicated surgery, temporary postsurgical urinary incontinence and newly developed freezing episodes postsurgically. Therefore, post-operative driving in such patients with DBS should also be particularly observed.

To date, there is lack of a single parameter or a test battery reliably indicating insufficient driving ability^5^. Recent own findings suggest a comprehensive test battery including the Montreal Cognitive Assessment (MoCA), TAP-M “flexibility” subscore (“Testbatterie für Aufmerksamkeitsprüfung”), Trail Making Test part A and Driving Behaviour Questionnaire (DBQ) subscore “errors“ as potential screening tools to detect PD patients at risk for driving^23^. However, this is not practicable in daily routine. Furthermore, as a selective decrease in frontal cognitive functions after STN-DBS has been described^24^, other frontal assessments might be a potential predictor. However, we did not find the Frontal Assessment Battery (FAB) predictive for bad driving in patients with PD in a previous study^23^.

We therefore suggest to advise patients with aspects of unsafe driving, for example based on information from a third party such as relatives, to have their ability to drive checked in general^5^ and especially after DBS surgery (optimally on-road by a professional driving instructor).

It is of note for the daily clinical routine, that postsurgical driving safety was found unaltered in the vast majority of our DBS patients, indicating that in general patients with DBS should not be advised differently regarding driving compared to other PD patients with similar clinical characteristics. However, future research should focus on detection of the only few but very relevant patients “at risk” for driving performance deterioration after DBS.

Our study has some limitations. First, we faced some rarely, randomly occurring technical problems in some patients regarding synchronization between INTERACT™ and DataView®. Therefore, two investigators simultaneously checked all logfiles manually and single missing data had to be interpolated. Randomly short stutters of the screen presenting the driving scenery occurred in some trials due to reduced storage capacity which might have confused some participants. Further studies should apply extended automatic error detection software such as we described elsewhere^23^ to avoid manual rater interaction as far as possible. Second, we initially planned two Baseline and two Follow-up investigations with identical driving routes and with averaged scores of both examinations to reduce bias due to a possible individual bad daily condition, especially in fluctuating patients. However, several participants performed only one session at Baseline and/or at Follow-up (reasons and distribution described in Supplementary Table 2 and Supplementary Table 3). In cases with two sessions mean scores were calculated. Despite a gap of a few days between investigations, a learning effect cannot be excluded in cases with two examinations at Baseline and/or Follow-up. However, numbers of DBS and nDBS patients with either two or only one examination at a time point were similar (Baseline PD-DBS vs. PD-nDBS: 5 vs. 7, Follow-up: 10 vs. 9). We think, a learning effect between Baseline and Follow-up investigations is unlikely because the mean time period between both investigations was 9 months. However, we did not test healthy subjects at Follow-up to control for this assumption. Future studies should provide randomly designed but in total identical challenges during the driving course. Third, comprehensive visual testing was not done but patients’ vision history had to be unsuspicious, and participants wore their appropriate visual aids.

Fourth, impairment of certain neuropsychological functions not detectable in the applied cognitive screening tests MMSE and PANDA might influence ability of on-road driving as well as driving in our simulator-setting that included several common driving challenges and distractors challenging executive functions. Further studies should focus on this aspect and aim to evaluate potential impairment of specific neuropsychological subdomains that might be related to driving deterioration after DBS surgery. Fifth, we did not quantify daytime sleepiness, which might have been different in patients undergone DBS compared to those who did not, mainly due to a reduction in sleep-inducing dopaminergic medication. However, sleepiness during simulator driving was neither reported by participants not recorded by the clinical observer. Furthermore, driving at Follow up was not superior in DBS compared to non-DBS patients, suggesting a potentially reduced daytime sleepiness in DBS subjects to be without relevant influence on driving simulator performance. Further studies should include a scale to quantify daytime sleepiness, e.g., the Epworth Sleepiness Scale^25^. Finally, despite advantages with respect to standardisation and replication^13^ and proof to reflect real life driving ability^2,13–15^, simulator testing likely cannot fully mimic real on-road driving feeling. However, to get as close as possible to that feeling we used a simulator model that fully complies with the European guideline 2003/59/EG and Driver Qualification Act.

To conclude, therapeutic intervention with subthalamic DBS does neither improve nor alter driving performance in the vast majority of PD patients but can deteriorate driving safety in single subjects. Unsafe driving prior to DBS surgery rather than certain clinical characteristics in candidates for STN-DBS might be a “predictive risk factor” for diminished driving safety postoperatively. Future research should focus on identification of those patients “at risk” for driving impairment after DBS implantation.

## Methods

### Participants

Participants were recruited in our Movement Disorders Centre (October 2013 to November 2017). Two groups of patients with PD that were comprehensively tested in-house for and rated as eligible for DBS^26,27^ were investigated and frequency-matched for age, sex/gender (self-reported) and cognition: (1) patients willing to undergo bilateral STN-DBS implantation (“PD-DBS”, implantations were performed in-house in all patients except one) and (2) patients who preferred to continue best medical treatment without DBS (“PD-nDBS”). Furthermore, a healthy control group of frequency-matched age without any neurologic diseases was recruited (“controls”) and assessed only once at Baseline to estimate the PD patients’ driving level in general. Further inclusion criteria were the possession of a valid driver license and driving actively on a regular base during the last three years. Exclusion criteria were the presence of aspects resulting in ineligibility for DBS, especially dementia or another relevant neurologic, psychiatric or cardiologic disease that might interfere with driving or result in consciousness disturbances (e.g. stroke, epilepsy etc.), an already known driving inability e.g. due to immobility or sleep attacks, a known higher order visual impairment or severe visual problems, the presence of hallucinations, unwillingness or inability to drive an automatic vehicle and orthopaedic symptoms interfering with handling of gas or brake pedals.

To exclude relevant cognitive impairment the Mini-Mental State Examination (MMSE^28^) was applied as screening tool with a cut-off score <24/30^29^. Additionally, we used the more sensitive and for PD validated Parkinson Neuropsychometric Dementia Assessment (PANDA^30^) to detect mild cognitive impairment^31^ and to evaluate subtle cognitive changes at Follow-up before and after DBS.

All participants were informed about the study verbally and by letter. Written informed consent was obtained from all participants. The study was approved by the local ethics committee of the Medical Council Hamburg (amendment to trial number PV3557).

### Data acquisition

First, demographic and clinical data of all participants were acquired including the patients’ MMSE and levodopa equivalent dosage (LED)^32^. All participants were asked about the date they had received their driver’s license, the mileage (in kilometres) they drove and number of accidents they had during the last three years and about a subjective rating on their individual driving safety.

**Fig. 3:**
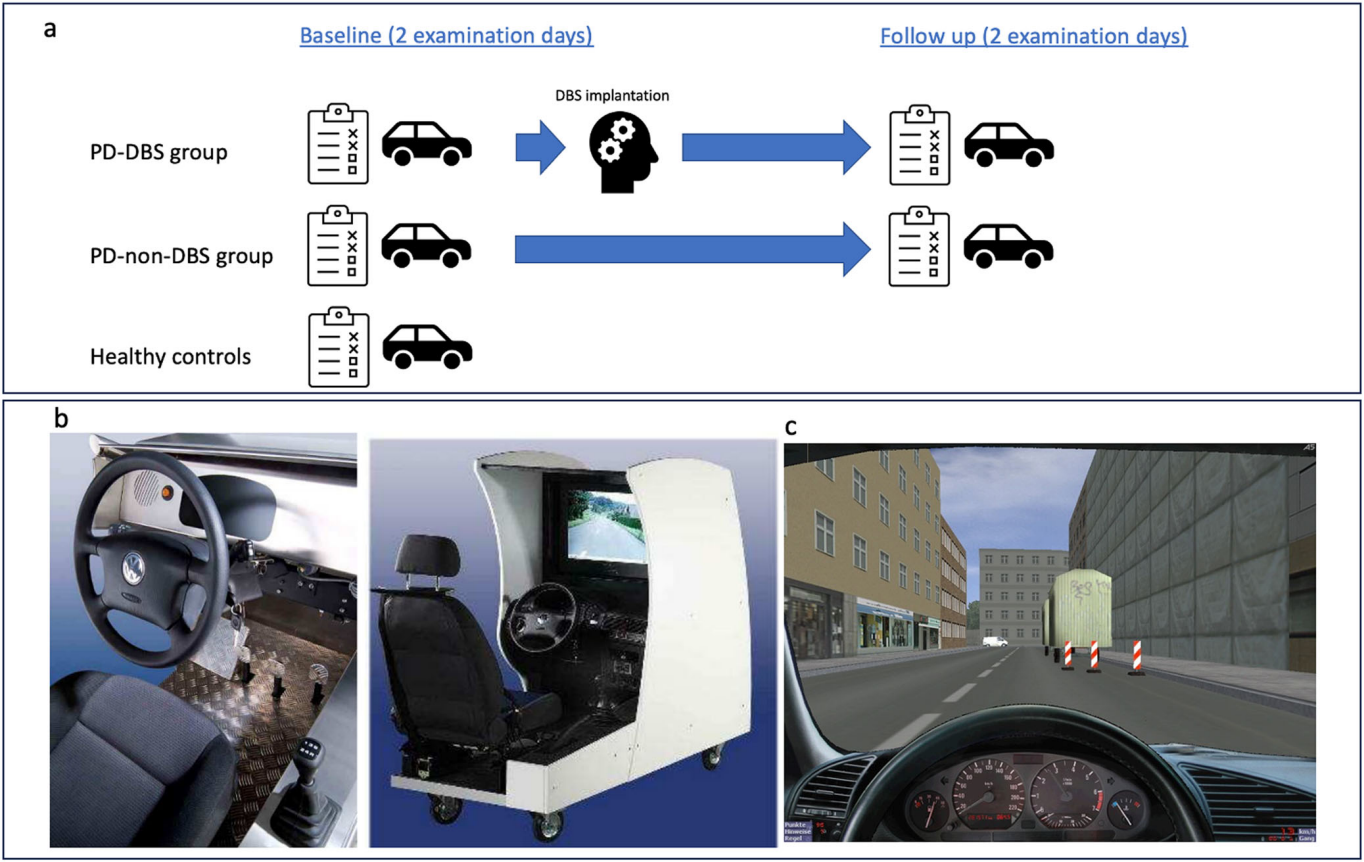
Study design and driving simulator system with software. **a** Study timeline for the three groups PD-DBS (Parkinson patients with DBS under regular medication), PD-nDBS (Parkinson patients without DBS under regular medication) and healthy controls. **b** Illustration of the driving simulator FT-SR 200 (Fa. SimuTech Gesellschaft für Fahrsimulation mbH, Bremen, Germany, http://www.simutech.de/index.htm) that was used in this study. **c** Screenshot of an inner city driving situation, software 3D Simulator driving-school (Besier 3D-Edutainment Wiesbaden, (c) Harro Besier 2001–2020, http://www.3Dfahrschule.de). The authors have the approval to use all pictures for this manuscript.

Both Parkinson’s disease groups (PD-DBS and PD-nDBS) were evaluated clinically and within the driving simulator in the clinical “on” state about one hour after taking their regular medication at two different time points (Fig. 3a): at Baseline and six to twelve months later, i.e. postoperatively in one group (Follow-up). It was planned that Baseline and Follow-up investigations include two separate examinations each on two different days with intervals no larger than three days (= four driving examinations in total).

Motor scores (Unified Parkinson’s Disease Rating Scale part III (UPDRS III))^33^ and Hoehn & Yahr Score (H&Y)^34^ were applied at all examination appointments. Participants’ cognition according to the PANDA, level of depression (Beck Depression Inventory; BDI)^35^ and quality of life (Parkinson’s Disease Questionnaire; PDQ-39)^36^ were tested once at Baseline and once at Follow-up. Controls were only evaluated once (Baseline). All participants were told to wear their regular vision aids best suitable for filling-out questionnaires and/or driving (near-vision or distance glasses, respectively).

### Driving simulator

We used a driving simulator FT-SR 200 (Fig. 3b; Fa. SimuTech GmbH, Bremen, Germany, http://www.simutech.de/index.htm), which is composed of an LCD panel and original VW Golf parts (2007) with automatic transmission comprising a detailed replication of the gearshift and pedals and a robust electronic engine to recreate steering forces. It complies with the European guideline 2003/59/EG and Driver Qualification Act (“Kraftfahrerqualifikationsgesetz”). Driving was simulated applying the software “3D-Simulator”, version 5.1 (Besier 3D-Edutainment Wiesbaden, Germany, (c) Harro Besier 2001–2020, http://www.3dfahrschule.de/, Fig. 3c).

### Driving sessions

Participants performed a 5-minute-training session comprising driving on a virtual parking area to accustom to the simulator system and clarify questions regarding its handling. Afterwards, they drove a predefined route without time limits and with identical challenges for everyone including sceneries in and out of town and realistic everyday life situations with oncoming traffic, crossroads, roundabouts, turning left and right, overtaking, crossing pedestrians and hazardous situations (e.g., animals crossing). Participants were asked to drive “as usual” by following common traffic regulations. Recording started on the first acceleration and ended after completing the route indicated by target flags on the screen.

### Driving analysis

For recording and analysis of driving performance, two different software components were used and synchronized with each other:

- The software DataView® (http://qualistar.chauvin-arnoux.com/de/dataview) recorded and analyzed videos of the main driving sessions providing speed information.
- Other driving performance aspects (e.g. driving errors) were directly recorded by the simulator software “INTERACT”™ (https://www.mangold-international.com/de/produkte/software/verhalten-erforschen-mit-interact) that served as a control for personal rater analysis described below in cases of potential uncertainty of error definition.

As our gold standard, videos of every driving session were analyzed simultaneously by two raters (EB and CS) who evaluated driving performance by counting the participants driving errors. We used a rater-based error counting additionally to INTERACT™ as we analyzed a broader spectrum of errors that were not included in the software, and we defined some errors slightly differently to allow for error severity classification (e.g., speed definitions; Table 4). Furthermore, we recognized some rarely appearing software problems during test runs (e.g., randomly error-logfiles were not exactly synchronized with error events) which could only be controlled manually. Two raters were chosen to increase the detection rate and decrease the missing rate of errors. Error counts and rating of error severity was done as consensus decision of both raters.

**Table 4:** Catalogue of driving errors.

Error category	Severity (factor)	In town (speed limit 50 km/h)	Out of town (speed limit 100 km/h)
Speed errors	Slight (1)	DRIVING too SLOW: <40 km/h for 10 s or more	- DRIVING too SLOW: <50 km/h for 10 s or more (Exception: slowing down due to curves in the road is allowed) - DRIVING IN FOG: >50 km/h for 5 s or more - DRIVING too FAST: ≥10 km/h above speed limit for 10 s or more
	Moderate (2)	DRIVING too FAST: >10 km/h above speed limit for 10 s or more	DRIVING too FAST: ≥20 km/h above speed limit for 10 s or more
	Severe (4)	- DRIVING too FAST: >20 km/h above speed limit for 10 s or more - DECELERATING (to 0 km/h) without any reason - RUNNING PAST A BUS: >10 km/h	DECELERATING (to 0 km/h) without any reason
	Very severe (8)	DRIVING too FAST: >30 km/h above speed limit for 10 s or more	DRIVING too FAST: >30 km/h above speed limit for 10 s or more
Distance keeping errors	Slight (1)	Distance <5 m	Distance < 1/2 speedometer^a^ for 10 s or more
	Moderate (2)	Distance <3 m	Distance < 1/4 speedometer^a^
	Severe (4)	Distance <1 m	Distance < 1/5 speedometer^a^
	Very severe (8)	Briefly very close	Briefly very close
Lane keeping errors	Slight (1)	- One-lane roads: TO SWERVE ABOUT A ROAD within the road edge marking - Multi-lane roads: TO SWERVE ABOUT A ROAD while crossing the road edge marking left or right	
	Moderate (2)	Touching the road edge marking (=*if lane reaches the medial third of the screen*)	
	Severe (4)	- CROSSING the road edge marking on the right side - LEAVING the lane completely	
	Very severe (8)	CROSSING the road edge marking on the left side	
Traffic sign errors	Slight (1)	GREEN LIGHT flashing for > 3 s (delayed starting)	
	Moderate (2)	YELLOW LIGHT	
	Severe (4)	- RED LIGHT flashing for <1 s - NO STOP AT CROSSWALK (although pedestrians approached)	
	Very severe (8)	RED LIGHT flashing for >1 s	
Indicator errors	Slight (1)	- MISSED TURN INDICATOR: to turn off - MISSED TURN INDICATOR: after passing another car on the right side	
	Moderate (2)	- MISSED TURN INDICATOR: after passing another car on the left side (when passing another car it is expected to use the turn indicator twice: when passing another car and when returning into the initial lane) - MISSED TURN INDICATOR: when making a lane change	
Accidents	Severe (4)	With inanimate OBJECTS	
	Very severe (8)	With PEOPLE or ANIMALS	

Error categories (left column) and error severity (second column) are described in detail for driving in (third column) and out of town (fourth column). Error severity is scaled into four categories (slight, moderate, severe and very severe) each with a predefined “severity factor” (1, 2, 4 or 8, respectively) for later analysis of the driving safety score.

^a^Minimum distance should be a certain speedometer length e.g., when driving 100 km/h, minimum distance should be half as much, thus 50 metres.

Driving errors were classified into six “error categories” considering (1) speed, (2) keeping distance, (3) keeping lane, (4) following traffic signs, (5) indicator errors and (6) accident. Errors were ranked according to their severity as slight, moderate, severe or very severe (Table 4).

Total number of errors (error rate), driving time (mean duration of driving sessions), error rate per error category and error severity were recorded. Moreover, a “driving safety score” (DSS) was defined to reflect overall driving safety. The DSS displays that error severity is more safety relevant than error rate and was calculated as follows: Every error counted (across all error categories) was multiplied by a “severity factor (SF)” ranking the errors according to their severity as geometric sequence with a power of two due to safety risk (SF 1 for slight errors, SF 2 moderate, SF 4 severe and SF 8 very severe and/or fatal errors (accident with injury to people or extremely dangerous errors with high safety-critical relevance)). All ranked errors were added up to build the DSS. Due to participants’ or technical problems (see results) not all patients completed the scheduled two examinations each at Baseline and Follow-up. As consequence we either used the mean error numbers at Baseline and/or Follow-up (two examinations available) or the total number of errors (only one examination available). The same was applied to UPDRS III and H&Y scores.

### Statistical analysis

Data was collected and pre-analyzed using IBM software SPSS version 18 (https:// www.ibm.com/analytics/spss-statistics-software). Final analysis was performed with the open-source software R version 4.0.2 (2020–06–22, R Core Team, https://www.R-project.org).

Baseline and Follow-up clinical characteristics were summarized (separately) as number of patients (and %) for categorical variables and as mean, standard deviation and range for continuous variables and were compared between groups using Chi-square tests, t-test (two-sided) and one-way ANOVA F-tests, respectively. Group comparisons of within-group differences from Baseline to Follow-up were performed with ANCOVA (= difference between the group-specific mean changes from Baseline, adjusted for Baseline).

Driving parameters were compared between groups (PD-DBS vs. PD-nDBS vs. healthy controls) at Baseline and Follow-up and within PD groups between Baseline and Follow-up using a single generalized linear mixed model with negative binomial link for count models and normal link for scores.

Spearman correlations between Baseline DSS and Baseline age, sex/gender, disease duration, LED, H&Y, UPDRS III, PANDA, MMSE and BDI were estimated for both PD groups combined to assess associations between clinical characteristics and participants’ general driving performance.

The driving safety score (DSS) and overall error rate were defined as primary outcomes with the aim to evaluate if driving performance changes from Baseline compared to Follow-up especially after DBS surgery in the PD-DBS group. All other parameters were defined as secondary outcomes.

## Supplementary information

Supplemental material

Reporting Summery

## Data Availability

The data that support the findings of this study are available from the corresponding author, upon reasonable request (e.g., scientific research interest). Only anonymous, but no person-identifying data can be provided.
