# Supplementary material for: Prospective controlled study on the effects of deep brain stimulation on driving in Parkinson’s disease
Source: NPJ Parkinsons Dis. 2023 Jul 3;9:105. doi: 10.1038/s41531-023-00545-6 (PMC10315394; doi:10.1038/s41531-023-00545-6)
Supplement: Supplementary file 1 — Supplemental material [file 41531_2023_545_MOESM1_ESM.pdf]

Supplemental material

Supplementary Table 1: Reasons for drop-out or not exploitable data sets

Reason	***Number of participants***
Problems with further recruitment/accessibility of the participant or because the participant did not show up at neither of the two Baseline and/or Follow-up appointments	n=16
Technical problems with the driving simulator or with the recording software	n=8
Early withdrawal from the study because of personal reasons	n=7
Early withdrawal from the study because of simulator sickness (e.g. nausea, vomiting, circulation problems etc.)	n=6
Problems with the handling of the driving simulator	n=2
Missing clinical scores	n=1
Severe dyskinesia (person preferred to not repeat the test on another day)	n=1
Severe akinesia (person preferred to not repeat the test on another day)	n=1
Newly occurring hallucinations on the day of examination	n=1
**Total**	**n=43**

Supplementary Table 1: Reasons and number of participants are given for drop-outs or not exploitable data sets.

Supplementary Table 2: Reasons for incomplete, but still exploitable data sets

Reason	***Number of subjects***
Problems with recruitment/accessibility or non-appearance of the participant	n=18
Technical problems	n=6
Simulator sickness occurring at the second examination for the first time	n=1
**Total**	**n=25**

Supplementary Table 2: Reasons and number of participants are given for incomplete, but still exploitable data sets.

Supplementary Table 3: Completion rate per session and group

Group	***Baseline Session 1***	***Baseline Session 2***	***Follow-up Session 1***	***Follow-up Session 2***
PD-DBS	n=21	n=19	n=23	n=13
PD-nDBS	n=29	n=22	n=27	n=22
Healthy subjects	n=33	-	-	-

Supplementary Table 3: Number of participants per group is given for the completion rate of each of the two sessions at Baseline and Follow-up.

Supplementary Table 4: Date of Follow-up (Months after Baseline)

Date of Follow-up (Months after Baseline)											
	4 months	6 months	7 months	8 months	9 months	10 months	11 months	12 months	15 months	17 months	18 months
Number of Participants (both PD groups)	1	6	7	11	7	3	12	2	1	1	1
PD-DBS	-	-	4	3	3	3	5	2	1	1	1
PD-nDBS	1	6	3	8	4	-	7	-	-	-	-

Supplementary Table 4: Number of PD patients per group is shown for the duration (given as months after Baseline) after which they participated in the Follow-up session.

Supplementary Table 5: Changes of clinical parameters from Baseline to Follow-up (ANCOVA) – all patients

Clinical parameter	PD-DBS Diff (95% CI)<br>n=23	PD-nDBS Diff (95% CI)<br>n=29	Difference (95% CI)	p-value
**Levodopa Equivalent Dose (LED, mg)**	**-496.9 (-621.3, -372.6)**	**-10.2 (132.0, 111.5)**	**-486.7 (-660.8, -312.6)**	**p<0.001*** **
**LED of Dopamine Agonists (mg)**	**-203.6 (-255.6, -151.6)**	**-34.6 (-85.5, 16.4)**	**-169.1 (-242.3, -95.9)**	**p<0.001*** **
PANDA	0.2 (-1.8, 2.2)	2.4 (0.7, 4.2)	-2.2 (-4.9, 0.4)	p=0.100
BDI	-1.5 (-3.0, 0.1)	-0.6 (-2.1, 0.8)	-0.8 (-2.9, 1.3)	p=0.438
PDQ-39	-3.4 (-9.1, 2.3)	-2.4 (-7.5, 2.7)	-1.0 (-8.8, 6.8)	p=0.793
UPDRS III	-1.5 (-6.1, 3.2)	3.6 (-0.5, 7.6)	-5.0 (-11.2, 1.1)	p=0.107
**Hoehn & Yahr score**	**-0.2 (-0.4, 0.0)**	**0.1 (0.0, 0.3)**	**-0.3 (-0.5, 0.0)**	**p=0.027***

Supplementary Table 5: Changes of clinical parameters from Baseline to Follow-up (shown for all patients including the two outliers) presented as the difference between group-specific mean changes from Baseline, adjusted for Baseline (ANCOVA).

Supplementary Table 6: Overall observed error rates (and DSS) of "worst five" participants

Ranking	Worst five participants							
	**Baseline**				**Follow-up**			
	ID	Group	**Overall error rate (mean)**	DSS (mean)	ID	Group	**Overall error rate (mean)**	DSS (mean)
1	**34***	PD-DBS	47	103.5	**30***	PD-DBS	88	297
2	62	PD-nDBS	46.5	106.0	**34***	PD-DBS	70.5	294.5
3	45	PD-nDBS	42	106.0	12	PD-DBS	40	134
4	**30***	PD-DBS	37	91.0	28	PD-nDBS	35	75
5	28	PD-nDBS	36.5	78.0	62	PD-nDBS	33	91

Supplementary Table 6: The "Worst five" ranking of participants with the highest overall error rates at Baseline (left) and Follow-up (right) are given with ID, group affiliation, individual overall error rate and their driving safety score (DSS). Especially in the Follow-up, two patients (*) showed about twice as many errors as the other participants. These two had high error rates at both points in time, but outstandingly in the Follow-up.

Supplementary Table 7: Correlations of the Driving safety scores (DSS) with clinical parameters (all patients)

Parameters	PD-DBS (n=23) (r-value (CI))	PD-nDBS (n=29) (r-value (CI))	Total (both PD) (r-value (CI))
Age	0.383 (-0.034, 0.687)	0.285 (-0.091, 0.590)	0.332 (0.065, 0.554)
Disease duration	0.461 (0.049, 0.739)	0.257 (-0.121, 0.570)	0.356 (0.090, 0.575)
Levodopa Equivalent Dose (LED)	0.025 (-0.391, 0.433)	0.179 (-0.200, 0.512)	0.113 (-0.165, 0.375)
LED of Dopamine Agonists	0.036 (-0.382, 0.442)	-0.085 (-0.438, 0.553)	-0.020 (-0.291, 0.255)
Hoehn & Yahr	0.415 (0.003, 0.706)	0.234 (-0.145, 0.553)	0.382 (0.060, 0.551)
UPDRS III	0.360 (-0.073, 0.679)	0.176 (-0.203, 0.510)	0.276 (0.000, 0.512)
PANDA	-0.286 (-0.631, 0.154)	-0.118 (-0.464, 0.260)	-0.183 (-0.437, 0.097)
MMST	-0.513 (-0.764, -0.128)	0.069 (-0.305, 0.425)	-0.174 (-0.427, 0.104)
BDI	0.032 (-0.385, 0.439)	0.063 (-0.310, 0.420)	0.096 (-0.181, 0.360)
Kilometers driven during the last 3 years	-0.238 (-0.92, 0.193)	-0.133 (-0.477, 0.245)	-0.183 (-0.434, 0.094)
DSS at Follow-up	0.639 (0.308, 0.832)	0.522 (0.193, 0.746)	0.574 (0357, 0.732)

Supplementary Table 7: Correlations between the Driving safety scores (DSS) and clinical parameters at Baseline are shown for both PD groups separately including outliers (PD-DBS middle left and PD-nDBS middle right column) and taken together (right column). R-values are given with confidence intervals (CI).

Supplementary Table 8: Baseline clinical parameters of study participants excluding the two outliers

**Clinical parameter**	**PD-DBS (n=21)**<br><br>**Mean +/- SD, Min-Max**	**PD-nDBS (n=29)**<br><br>**Mean +/- SD, Min-Max**	**Healthy (n=33)**<br><br>**Mean +/- SD, Min-Max**	**Statistics (p-value)**
Age (years)	59.1 (+/- SD 7.7; 45-71)	61.4 (+/- SD 7.6; 49-73)	57.7 (+/- SD 11.3; 29-77)	F(2,80)=1.209, p=0.304
Gender (number of female/male)	6 /15	5/24	12/21	$\chi^2(2)$=2.828 ,p=0.243[1]
Disease duration (years)	10.9 (+/- SD 5.0; 5-27)[5]	9.0 (+/- SD 4.8; 2-24)	n.a.	F(1,47)=1.793, p=0.187
Ownership of driver license (years)	40.3 (+/- SD 10.1; 20-58)	41.1 (+/- SD 7.8; 26-55)	37.7 (+/- SD 11.7; 11-58)	F(2,80)=0.976, p=0.381
Kilometers driven during the last 3 years? (km)	32,471 (+/- SD 26,320; 300-80,000)	34,293(+/- SD 59,797; 1000-30,000)	41,424 (+/- SD 42,5672; 2000-20,000)	F(2,80)=0.279, p=0.744
Subjective Driving Safety (number of participants per choice)	- safe: 11<br>- average: 8<br>- unsafe: 2	- safe: 15<br>- average: 11<br>- unsafe: 3	- safe: 20<br>- average: 11<br>- unsafe: 2	$\chi^2(4)$=0.762, p=0.943[1]
Accidents during last 3 years (number of participants per choice)	- 1 accident: 3<br>- >1 accident: 0	- 1 accident: 8<br>- >1 accident: 0	- 1 accident: 3<br>- >1 accident: 2	$\chi^2(4)$=6.654, p=0.155[1]
Levodopa Equivalent Dose (LED, mg)	1,172.6 (+/- SD 623.0; 152-2,740)	1,070.6 (+/- SD 455.1; 210-1,812)	n.a.	F(1,48)=0.449, p=0.506

Dopamine Agonists - LED (mg)	283.4 (+/- SD 225.2; 0-880)	347.3 (+/- SD 247.5; 0-1280)	n.a.	F(1,48)=1.941, p=0.170
Dopamine Agonists – Frequency of use (n)	18	28	n.a.	$\chi^2(1)=1.944$, p=0.163[1]
H&Y	2.1 (+/- SD 0.7; 0-3.5)	2.3 (+/- SD 0.6; 1-3)	n.a.	F(1,48)=-1.068, p=0.291
UPDRS III	15.6 (+/- SD 10.3; 1.5-38.5)	16.4 (+/- SD 8.5; 3.5-39.5)	n.a.	F(1,48)=-0.293, p=0.771
PANDA	20.7 (+/- SD 6.4; 9-30)[2]	20.2 (+/- SD 6.0; 5-28)	24.8 (+/- SD 4.2; 14-30)	**F(2,79)=6.343, p=0.003** **
MMSE	28.3 (+/- SD 3.3; 15-30)	28.7 (+/- SD 1.9; 24-30)	28.8 (+/- SD 1.5; 24-30)	F(2,80)=0.355, p=0.702
BDI	9.4 (+/- SD 7.1; 1-30)	7.0 (+/- SD 5.2; 1-25)	2.2 (+/- SD 2.8; 0-13)[3]	**F(2,79)=14.183, p<0.001*** **
PDQ-39	30.7 (+/- SD 18.9; 6.4-82.0)	25.1 (+/- SD 11.8; 5.1-53.2)	2.2 (+/- SD 3.5; 0-12.8)[4]	**F(2,78)=43.125, <0.001*** **

Supplementary Table 8: Clinical parameters and scores at Baseline appointment are shown for the three groups excluding the two PD-DBS outliers as mean (standard deviation SD; minimum and maximum). P-values of univariate ANOVA are given in the last column. [1]Analyzed with chi-square test (Pearson). [2]Data of 1 participant not documented (PANDA). [3]Data of 1 participant not documented (BDI). [4]Data of 2 participants not documented (PDQ-39). [5]Data of 1 participant missing (disease duration). n.a. = not applicable.

Supplementary Table 9: Follow-up clinical parameters of study participants excluding the two outliers

Clinical parameter	PD-DBS (n=21)<br><br>Mean +/- SD, Min-Max	Number of patients with available data	PD-nDBS (n=29)<br><br>Mean +/- SD, Min-Max	Number of patients with available data	Statistics (p-value)
Levodopa Equivalent Dose (LED, mg)	675.7 (+/- SD 367.0; 0-1322.5)	n=21	1111.4 (+/- SD 504.0; 75-1948.8)	n=24	**t(43)=-3.272, p=0.002***
Dopamine Agonists - LED (mg)	103.5 (+/- SD 118.3; 0-484.8)	n=21	309.7 (+/- SD 205.4; 0-800)	n=24	**t(43)=-4.074, p<0.001****
Dopamine Agonists – Frequency of use (n)	14	n=21	22	n=24	**$\chi^2(1)=4.375$, p=0.036*[1]**
H&Y	2.0 (+/- SD 0.5; 1-3)	n=21	2.3 (+/- SD 0.4; 1.25-3)	n=29	**t(48)=-2.316, 0.025***
UPDRS III	15.7 (+/- SD 10.5; 4-47)	n=21	20.2 (+/- SD 9.7; 8-48)	n=29	t(48)=-1.566, 0.124
PANDA	21.1 (+/- SD 6.2; 9-29)	n=21	22.7 (+/- SD 4.6; 11-28)	n=29	t(48)=-1.048, p=0.300
BDI	6.8 (+/- SD 4.2; 1-16)	n=18	6.6 (+/- SD 4.9; 0-20)	n=22	t(38)=0.166, p=0.869
PDQ-39	26.6 (+/- SD 13.2; 4.2-46.5)	n=15	24.8 (+/- SD 13.4; 10.9-52.8)	n=21	t(34)=0.394, p=0.696

Supplementary Table 9: Clinical parameters and scores at Follow-up appointment are shown for both the PD groups as mean (standard deviation SD; minimum and maximum). The two outliers of the PD-DBS group are excluded. P-values of t-tests are given in the last column. Because at Follow-up some patients' data is missing, we included the number of PD patients with complete datasets per clinical parameter. [1]Calculated with Pearson chi-square test.

Supplementary Table 10: Changes of clinical parameters from Baseline to Follow-up (ANCOVA) – outliers excluded

Clinical parameter	PD-DBS Diff (95% CI)<br><br>n=21	PD-nDBS Diff (95% CI)<br><br>n=29	Difference (95% CI)	p-value
**Levodopa Equivalent Dose (LED, mg)**	**-484.0 (-616.2, -351.8)**	**-10.2 (-133.9, 113.4)**	**-473.8 (-655.0, -292.7)**	**p<0.001*****
**LED of Dopamine Agonists (mg)**	**-202.3 (-257.9, -146.7)**	**-33.9 (-85.9, 18.0)**	**-168.4 (-244.9, -91.8)**	**p<0.001*****
PANDA	0.3 (-1.8, 2.5)	2.4 (0.6, 4.2)	-2.0 (-4.8, 0.8)	p=0.152
BDI	-1.3 (-2.9, 0.3)	-0.6 (-2.0, 0.9)	-0.8 (-2.9, 1.4)	p=0.489
PDQ-39	-4.7 (-10.8, 1.5)	-2.4 (-7.6, 2.8)	-2.3 (-10.5, 5.9)	p=0.578
UPDRS III	-0.4 (-5.2, 4.3)	4.0 (-0.0, 8.0)	-4.4 (-10.7, 1.8)	p=0.159
Hoehn & Yahr score	-0.1 (-0.3, 0.1)	0.2 (0.0, 0.3)	-0.2 (-0.5, 0.0)	p=0.069

Supplementary Table 10: Changes of clinical parameters from Baseline to Follow-up (shown for the participants after excluding the two outliers) presented as the difference between group-specific mean changes from Baseline, adjusted for Baseline (ANCOVA).

Supplementary Table 11: Driving performance parameters of remaining subjects (excluding outliers)

					Between group comparisons				Within group comparisons			
**Parameter**	**Time point**	**Controls (n=33)**	**PD-DBS (n=21)**	**PD-nDBS (n=29)**	**PD vs Ctrls**	**p-value**	**DBS vs nDBS**	**p-value**	**FU vs BL: DBS**	**p-value**	**FU vs BL: nDBS**	**p-value**
**Driving time (in s; estimated residuals, LMM)**	Baseline	835.0 (788.9, 881.1)	843.4 (785.7, 901.2)	856.9 (807.8, 906.1)	15.19 (-44.50, 74.87)	0.615	-13.52 (-89.37, 62.34)	0.725	-	-	-	-
	Follow-up	-	801.3 (743.6, 859.1)	831.7 (780.8, 882.6)	-	-	-30.34 (-107.32, 46.65)	0.437	-42.10 (-115.48, 31.29)	0.258	-25.28 (-89.10, 38.54)	0.435
**Absolute Error rate (mean, estimated residuals, LMM)**	Baseline	15.9 (13.9, 18.1)	17.8 (15.2, 20.9)	19.0 (16.6, 21.7)	1.16 (0.98, 1.37)	0.086	0.94 (0.76, 1.16)	0.545	-	-	-	-
	Follow-up	-	17.5 (14.9, 20.5)	17.4 (15.2, 20.0)	-	-	1.00 (0.81, 1.24)	0.988	0.98 (0.84, 1.14)	0.808	0.92 (0.81, 1.04)	0.195

**Error severity (quotients, gLMM)**												
Slight	Baseline	9.4<br>(8.3, 10.7)	9.2<br>(7.8, 10.8)	10.1<br>(8.9, 11.6)	1.02<br>(0.87, 1.21)	0.775	0.90<br>(0.73, 1.12)	0.352	-	-	-	-
	Follow-up	-	9.3<br>(7.9, 11.0)	9.0<br>(7.8, 10.3)	-	-	1.04<br>(0.84, 1.29)	0.739	1.01<br>(0.83, 1.24)	0.900	0.88<br>(0.75, 1.04)	0.144
Moderate	Baseline	3.7<br>(2.9, 4.7)	5.7<br>(4.3, 7.5)	5.4<br>(4.3, 6.9)	1.51<br>(1.12, 2.05)	**0.008**	1.05<br>(0.73, 1.51)	0.777	-	-	-	-
	Follow-up	-	5.6<br>(4.2, 7.4)	5.5<br>(4.4, 7.0)	-	-	1.01<br>(0.71, 1.46)	0.942	0.98<br>(0.77, 1.26)	0.897	1.02<br>(0.83, 1.26)	0.833
Severe	Baseline	1.8<br>(1.4, 2.4)	1.5<br>(1.1, 2.2)	1.7<br>(1.3, 2.3)	0.87<br>(0.62, 1.22)	0.427	0.89<br>(0.57, 1.40)	0.618	-	-	-	-
	Follow-up	-	1.2<br>(0.8, 1.8)	1.5<br>(1.1, 2.0)	-	-	0.83<br>(0.51, 1.35)	0.440	0.81<br>(0.48, 1.37)	0.433	0.88<br>(0.58, 1.33)	0.535
Very severe	Baseline	0.7<br>(0.5, 1.1)	1.1<br>(0.7, 1.7)	1.3<br>(0.9, 2.0)	1.62<br>(0.97, 2.73)	0.066	0.80<br>(0.44, 1.45)	0.456	-	-	-	-
	Follow-up	-	1.0<br>(0.6, 1.6)	1.0<br>(0.7, 1.6)	-	-	0.98<br>(0.53, 1.82)	0.951	0.95<br>(0.55, 1.63)	0.839	0.77<br>(0.49, 1.20)	0.245
**Error classes (quotients, gLMM)**												
Distance	Baseline	0.6<br>(0.2, 2.2)	1.4<br>(0.3, 6.1)	1.7<br>(0.5, 5.9)	2.39<br>(0.50, 11.37)	0.272	0.83<br>(0.12, 5.75)	0.845	-	-	-	-
	Follow-up	-	1.0<br>(0.2, 4.7)	1.3<br>(0.4, 4.7)	-	-	0.79<br>(0.11, 5.58)	0.811	0.76<br>(0.09, 6.22)	0.795	0.79<br>(0.13, 4.70)	0.798
Indicator	Baseline	4.9<br>(4.0, 6.1)	6.6<br>(5.2, 8.4)	6.0<br>(4.9, 7.5)	1.28<br>(0.99, 1.66)	0.061	1.10<br>(0.80, 1.51)	0.570	-	-	-	-
	Follow-up	-	5.3<br>(4.1, 6.8)	5.1<br>(4.1, 6.4)	-	-	1.03<br>(0.74, 1.44)	0.854	0.80<br>(0.60, 1.08)	0.145	0.85<br>(0.66, 1.10)	0.221
Lane keeping	Baseline	8.7<br>(6.3, 12.1)	12.5<br>(8.4, 18.5)	13.0<br>(9.3, 18.2)	1.46<br>(0.97, 2.21)	0.071	0.96<br>(0.57, 1.60)	0.867	-	-	-	-
	Follow-up	-	13.4<br>(9.1, 19.9)	12.3<br>(8.8, 17.2)	-	-	1.09<br>(0.65, 1.82)	0.738	1.08<br>(0.76, 1.53)	0.677	0.94<br>(0.70, 1.28)	0.712
Accident	Baseline	2.5<br>(1.5, 4.4)	3.1<br>(1.6, 6.3)	3.3<br>(1.8, 5.9)	1.27<br>(0.62, 2.60)	0.515	0.95<br>(0.38, 2.34)	0.910	-	-	-	-
	Follow-up	-	1.7<br>(0.8, 3.5)	2.8<br>(1.5, 5.0)	-	-	0.62<br>(0.24, 1.58)	0.315	0.55<br>(0.20, 1.48)	0.232	0.83<br>(0.36, 1.91)	0.665
Velocity (Speed)	Baseline	9.6<br>(8.4, 11.0)	8.1<br>(6.8, 9.7)	8.9<br>(7.7, 10.4)	0.88<br>(0.74, 1.06)	0.180	0.91<br>(0.72, 1.15)	0.425	-	-	-	-
	Follow-up	-	8.4<br>(7.0, 10.0)	8.7<br>(7.5, 10.1)	-	-	0.96<br>(0.76, 1.22)	0.749	1.03<br>(0.81, 1.31)	0.803	0.97<br>(0.80, 1.19)	0.793
Traffic sign	Baseline	3.2<br>(2.1, 4.7)	3.7<br>(2.3, 6.0)	5.3<br>(3.6, 7.9)	1.41<br>(0.86, 2.33)	0.175	0.70<br>(0.37, 1.30)	0.254	-	-	-	-
	Follow-up	-	1.9<br>(1.1, 3.2)	3.4<br>(2.3, 5.2)	-	-	0.56<br>(0.28, 1.09)	0.085	0.51<br>(0.25, 1.05)	0.067	0.64<br>(0.36, 1.14)	0.131
**Driving safety score (DSS, quotients, gLMM)**	Baseline	31.9<br>(25.4, 38.4)	38.3<br>(30.2, 46.5)	42.3<br>(35.4, 49.3)	8.43<br>(0.04, 16.82)	**0.049**	-4.01<br>(-14.68, 6.65)	0.458	-	-	-	-
	Follow-up	-	36.5<br>(28.4, 44.7)	37.7<br>(30.8, 44.6)	-	-	-1.14<br>(-11.81, 9.52)	0.833	-1.79<br>(-8.85, 5.27)	0.618	-4.66<br>(-10.66, 1.35)	0.128

Supplementary Table 11: Driving performance parameters are presented (excluding outliers) for each of the three groups (PD-DBS = PD patients with DBS; PD-nDBS = PD patients without DBS; Ctrl = healthy controls) at Baseline (upper row) and Follow-up (bottom row) as estimated residuals (LMM) or quotients (gLMM). Results of between-group (left) and within-group (right) comparisons are given as p-values. Significant p-values are marked bold. Statistical results (p-values) varying from main analysis (including outliers) are marked in yellow for a better understanding.

Supplementary Table 12: Correlations of the Driving safety scores (DSS) with clinical parameters (outliers excluded)

Parameters	PD-DBS (n=21) (r-value (CI))	PD-nDBS (n=29) (r-value (CI))	Total (both PD) (r-value (CI))
Age	0.204 (-0.250, 0.584)	0.285 (-0.091, 0.590)	0.270 (-0.009, 0.510)
Disease duration	0.578 (0.182, 0.813)	0.257 (-0.121, 0.570)	0.370 (0.099, 0.590)
Levodopa Equivalent Dose (LED)	0.038 (-0.401, 0.462)	0.179 (-0.200, 0.512)	0.112 (-0.172, 0.379)
LED of Dopamine Agonists	-0.024 (-0.451, 0.412)	-0.085 (-0.438, 0.291)	-0.035 (-0.310, 0.246)
Hoehn & Yahr	0.215 (-0.239, 0.591)	0.234 (-0.145, 0.553)	0.245 (-0.036, 0.490)
UPDRS III	0.264 (-0.185, 0.624)	0.176 (-0.203, 0.510)	0.237 (-0.045, 0.483)
PANDA	-0.250 (-0.624, 0.216)	-0.118 (-0.464, 0.260)	-0.163 (-0.425, 0.124)
MMST	-0.374 (-0.624, 0.069)	0.069 (-0.305, 0.425)	-0.103 (-0.371, 0.181)
BDI	-0.036 (-0.461, 0.402)	0.063 (-0.310, 0.420)	0.047 (-0.235, 0.321)
Kilometers driven during the last 3 years	-0.256 (-0.619, 0.197)	-0.133 (-0.477, 0.245)	-0.204 (-0.456, 0.079)
DSS at Follow-up	0.527 (0.123, 0.781)	0.522 (0.193, 0.746)	0.522 (0.285, 0.699)

Supplementary Table 12: Correlations between the Driving safety scores (DSS) and clinical parameters at Baseline are shown for both PD groups separately excluding outliers (PD-DBS middle left and PD-nDBS middle right column) and taken together (right column). R-values are given with confidence intervals (CI).

Supplementary Table 13: Clinical parameters of the two outliers

Clinical parameter	Outlier 1 (ID 30)		Outlier 2 (ID 34)	
	Baseline	Follow-up	Baseline	Follow-up
Age	72		70	
Gender	Female		male	
Disease duration	16 years		7 years	
Ownership of driver license	52 years		52 years	
Kilometers driven during the last 3 years? (km)	30.000		20.000	
Subjective Driving Safety	"average"		"average"	
Accidents during last 3 years	0		0	
Levodopa Equivalent Dose (mg)	1115.3	440.0	1300	600
Dopamine agonist	Yes	Yes	Yes	No
H&Y	Session 2: 4	Session 1: 2	Session 1: 3<br>Session 2: 4<br>Mean: 3.5	Session 1: 2.5<br>Session 2: 2<br>Mean: 2.5
UPDRS III	Session 2: 17	Session 1: 6	Session 1: 40<br>Session 2: 46<br>Mean: 43	Session 1: 5<br>Session 2: 10<br>Mean: 8
PANDA	24	17	10	17
MMSE	27	29	26	26
BDI	10	10	10	4
PDQ_39	21.1	27.3	31.4	38.2
Time between driving sessions	7 months		8 months	
Time from Baseline investigation to DBS surgery	1 week		4 weeks	
Specifics of surgery	Surgery awake; surgical complication with iatrogenic motor-guided placement of microelectrode 3 cm below target point on left side, clinically no obvious side effects,		Surgery awake; intraoperatively increasing exhaustion and psychomotor restlessness: need to change to general anesthesia; postsurgical temporary urinary incontinence	

	minor bleeding above target point; postsurgical temporary urinary incontinence			
Specifics of DBS programming	Sufficient suppression of sweating, fluctuations and tremor under “standard” stimulation parameters; reduction of medication		After vanishing of marked postoperative stun effect of clinical symptoms: very narrow therapeutic stimulation window with respect to inducing mania and ICD (aggressivity, binge eating) in 3 of 4 lower contacts	
Clinical specifics at time of testing	Moderate, not functionally disabling dyskinesia	Appeared slightly irritated and disorganized	Could not relax, tense posture while driving	None
Improvement of UPDRS III in presurgical Levodopa challenge test	25/17 (32%)		55/40 (27.3%)	
Clinical specifics before (around Baseline) and after (Follow-up) surgery according to medical records	H&Y 4/3 (off/on); severe motor fluctuations, dyskinesia, off foot dystonia	Sufficient effect on fluctuations and dyskinesia, but new occurrence of freezing episodes with rare falls	H&Y 4/3 (off/on); wheeled walker, slight motor fluctuations, tremor; marked impairment of finger dexterity; relatives suggested to quit car driving	Marked clinical improvement of fluctuations and tremor, but new occurrence of freezing episodes and festination without falls; frequently bike cycling up to 30km; undergone voluntary driving test with a driving-school instructor without relevant objection; rated as fit for driving by driving instructor and neurologist
Supplemental neuropsychologic specifics according to medical records	MATTIS 137/144	Four months after surgery but 3 months prior to Follow-up	MATTIS 139/144<br><br>MOCA 28/30	Six months after Follow-up investigation:

	MOCA 29/30	investigation:<br><br>PANDA 19/30<br><br>Unsuspicious HADS (A7,D6)<br><br>Unsuspicious observation of behavior, but in conversation references to impairment of mnestic functions		PANDA 21/30<br><br>Unsuspicious HADS (A7, D6)<br><br>Unsuspicious observation of behavior, but in conversation references to impairment of mnestic functions (did not remember presurgical testing by identical rater)
Error rate per session	Session 2: 37	Session 1: 88	Session 1: 52 Session 2: 42 Mean: 47	Session 1: 84 Session 2: 57 Mean: 70.5
DSS per session	Session 2: 91	Session 1: 297	Session 1: 115 Session 2: 92 Mean: 103.5	Session 1: 373 Session 2: 216 Mean: 294.5
Driving time per session (in s)	Session 2: 943	Session 1: 1265	Session 1: 975 Session 2: 926 Mean: 950.5	Session 1: 980 Session 2: 814 Mean: 897
Duration between Baseline and Follow-up (in months)	7		8	

Supplementary Table 13: Clinical parameters of the two outliers with very high error rates at Baseline and Follow-up. Abbreviation: n.i.= no information.
